# Extranuclear effects of thyroid hormones and analogs during development: An old mechanism with emerging roles

**DOI:** 10.3389/fendo.2022.961744

**Published:** 2022-09-23

**Authors:** Sandra Incerpi, Fabio Gionfra, Roberto De Luca, Elena Candelotti, Paolo De Vito, Zulema A. Percario, Stefano Leone, Davide Gnocchi, Miriam Rossi, Francesco Caruso, Sergio Scapin, Paul J. Davis, Hung-Yun Lin, Elisabetta Affabris, Jens Z. Pedersen

**Affiliations:** ^1^ Department of Sciences, University Roma Tre, Roma, Italy; ^2^ Beth Israel Deaconess Medical Center, Harvard Medical School, Boston, MA, United States; ^3^ Department of Biology, University Tor Vergata, Rome, Italy; ^4^ Interdisciplinary Department of Medicine, University of Bari, School of Medicine, Bari, Italy; ^5^ Department of Chemistry, Vassar College, Poughkeepsie, NY, United States; ^6^ Department of Cellular and Developmental Biology, Sapienza University, Rome, Italy; ^7^ Department of Medicine, Albany Medical College, Albany, NY, United States; ^8^ Pharmaceutical Research Institute, Albany College of Pharmacy and Health Sciences, Albany, NY, United States; ^9^ Taipei Cancer Center, Taipei Medical University, Taipei, Taiwan; ^10^ Graduate Institute of Cancer Biology and Drug Discovery, College of Medical Science and Technology, Taipei Medical University, Taipei, Taiwan; ^11^ Traditional Herbal Medicine Research Center of Taipei, Medical University Hospital, Taipei Medical University, Taipei, Taiwan; ^12^ TMU Research Center of Cancer Translational Medicine, Taipei Medical University, Taipei, Taiwan

**Keywords:** thyroid hormone, 3,5-diiodothyronine, integrin αvβ3, Na/K-ATPase, cancer, virus infection, signal transduction, gibberellins

## Abstract

Thyroid hormones, T_3_ (triiodothyronine) and T_4_ (thyroxine), induce a variety of long-term effects on important physiological functions, ranging from development and growth to metabolism regulation, by interacting with specific nuclear or cytosolic receptors. Extranuclear or nongenomic effects of thyroid hormones are mediated by plasma membrane or cytoplasmic receptors, mainly by αvβ3 integrin, and are independent of protein synthesis. A wide variety of nongenomic effects have now been recognized to be elicited through the binding of thyroid hormones to this receptor, which is mainly involved in angiogenesis, as well as in cell cancer proliferation. Several signal transduction pathways are modulated by thyroid hormone binding to αvβ3 integrin: protein kinase C, protein kinase A, Src, or mitogen-activated kinases. Thyroid hormone-activated nongenomic effects are also involved in the regulation of Na^+^-dependent transport systems, such as glucose uptake, Na^+^/K^+^-ATPase, Na^+^/H^+^ exchanger, and amino acid transport System A. Of note, the modulation of these transport systems is cell-type and developmental stage-dependent. In particular, dysregulation of Na^+^/K^+^-ATPase activity is involved in several pathological situations, from viral infection to cancer. Therefore, this transport system represents a promising pharmacological tool in these pathologies.

## Introduction

Thyroid hormones, triiodothyronine (T_3_) and thyroxine (T_4_), are key hormones, involved in the control of fundamental physiologic functions, ranging from development, metabolism, thermic homeostasis and cognitive functions (1). In the last two decades, relevant physiological roles have been ascribed also to several thyroid hormone metabolites that previously were considered without any function, such as diiodothyronines and monoiodothyronines, as reviewed elsewhere (2). Also, non-nuclear, nongenomic, short-term effects of thyroid hormones and thyroid hormone metabolites have been described associated with important physiological roles (3).

Here, we take a snapshot on the role of nongenomic effects of thyroid hormones and of one of their metabolites, 3,5-diiodothyronine (3,5-T_2_), in particular focusing on the relevance for tumor biology and immune function.

## Genomic and nongenomic actions of thyroid hormones

Genomic effects of thyroid hormones, T_3_ and T_4_, regulate many steps of metabolism, growth and development. Such nuclear effects occur after the binding of T_3_ to thyroid hormone receptors, TRα and TRβ, which are members of the nuclear receptor superfamily. Thyroid hormone receptors bind DNA at the thyroid hormone response elements (TREs), mainly as homodimers, but also as heterodimers, in particular with retinoid X receptor (RXR) or the retinoic acid receptor (RAR; 1). Nuclear factors called thyroid hormone receptor-associated proteins enhance the binding of thyroid hormone receptor to TREs, while co-repressor proteins bind the unliganded receptors and directly inhibit basal transcription. The co-repressors NCoR (nuclear receptor co-repressor 2) and SMRT (Silencing Mediator for Retinoid and Thyroid hormone receptors) also recruit histone deacetylases. Binding of T_3_ to its receptor site induces a conformation change that leads to the dissociation of corepressors and the recruitment of coactivators, and thus initiates the ligand-induced transcriptional activity (1, 4).

In addition to genomic responses, thyroid hormones also elicit nongenomic effects, which typically are initiated at the plasma membrane or cytoplasm level. These effects are characterized by a time-course of seconds to minutes, and do not rely on the interaction with the nuclear receptors. The receptor protein differs according to the cell type and it can be nuclear or cytosolic (5–14). The αvβ3 integrin acts as a thyroid hormone receptor on the cell membrane, and many rapid effects have been reported to be mediated by this integrin (15). The downstream signaling pathway involves mitogen-activated protein kinase (MAPK, ERK1/2) or phosphatidylinositol 3-kinase (PI3K), and can result in the stimulation of angiogenesis and of tumor cell growth (15–18). Integrin αvβ3 presents two binding sites for thyroid hormones: T_3_ binds to the S1 site activating Src kinase, which then triggers PI3K downstream signaling, leading to translocation of cytoplasmic TRα to the nucleus and activation of the gene hypoxia inducible factor-1α (*HIF-1α*). These effects are inhibited by the tripeptide arginine-glycine-aspartate (RGD), a ligand domain for several integrins, and by tetraiodothyroacetic acid (tetrac), a product of thyroid hormone metabolism, considered a probe for the involvement of αvβ3 integrin. Both T_3_ and T_4_ bind to the second integrin site S2, with T_4_ being more efficient than T_3_, leading to the activation of ERK1/2, which results in the nuclear translocation of TRβ1 and in tumor cell proliferation. The effect of T_4_ can be blocked by the MEK1/2 inhibitor PD98059, and also in this case hormone binding to αvβ3 integrin is directly inhibited by the RGD tripeptide and by Tetrac (19–22).

Interestingly, the transcription of some cytokines and chemokines, such as the fractalkine ligand (CX3CL1) and receptor (CX3CR1) genes, were reported to be initiated through the αvβ3 integrin, and to be downregulated by Tetrac in tumor cells (18, 23). Nongenomic effects of thyroid hormones at the plasma membrane level have been associated to membrane transport systems, such as glucose transport, the plasma membrane enzymes Na^+^/K^+^-ATPase, Na^+^/H^+^-exchanger, Ca^2+^-ATPase and the Na^+^-sensitive amino acid transport. The modulation of plasma membrane Na^+^/K^+^-ATPase activity was shown to be tissue and cell type-dependent (7, 9, 19, 24, 25).

## The chick embryo hepatocyte and a new hormone: 3,5-diiodothyronine (3,5-T_2_)

Chicken is a good model to study development and the effect of thyroid hormones (24). Differently to mammals, which have intrauterine development, chick embryo develops in a closed environment devoid of maternal endocrine influences. The levels of thyroid hormones are quite low during chick embryo development, but T_3_ increases at the time of pipping when the embryo opens the air chamber and shifts from allantoic to lung respiration. The values of T_3_ remain high until hatching (26). This is due to a delicate equilibrium between the activity of deiodinases D1 and D3. The D1 is for the outer ring deiodination, while D3 controls the inner deiodination and thus inactivation of T_3_. This modulation appears to be operative in the last days before and at the beginning of hatching, between 14^th^ and 17^th^ days of development. In particular, D1 activity increases and D3 activity decreases around hatching, and this results in a significant increase in the level of T_3_. 3,5-T_2_ is a metabolite that probably results from the deiodination of T_3_, (27). 3,5-T_2_ mimics some metabolic effects of thyroid hormones, and its plasma concentrations are in the picomolar range (28). 3,5-T_2_ increases the resting metabolic rate (RMR) as well as T_3_, but the effect is faster and not inhibited by actinomycin D (29). The 3,5-T_2_ increased survival of hypothyroid rats from long-term cold exposure, being very efficient in the stimulation of mitochondriogenesis (30). At the same time 3,5-T_2_ stimulated body weight loss when administered to high fat diet (HFD)-fed rats, without cardiotoxic effects (31). 3,5-T_2_ stimulates mitochondrial uncoupling, decreases ATP synthesis, and increases fat burning, thus antagonizing obesity (32). 3,5-T_2_ antilipidemic effects are mediated by two different pathways, AMPK and the deacetylase sirtuin 1 (SIRT1; 33). Of note, it was reported long-term administration to rats of 3,5-T_2_ resulted in suppressed thyroid function and central hypothyroidism (34).

Our previous observations prompted us to study the effects of 3,5-T_2_ during development in chick embryo hepatocytes in different membrane transport systems: Na^+^/H^+^-exchanger, Na^+^-dependent amino acid transport and Na^+^/K^+^-ATPase activity at different stages of development 14 and 19 days (9, 13, 14, 25).

## Na^+^/K^+^-ATPase

Na^+^/K^+^-ATPase, also called the Na^+^ pump, keeps a gradient of Na^+^ and K^+^ ions across the plasma membrane, by transporting three Na^+^ ions out and two K^+^ ions inside the cell against gradient at the expense of ATP hydrolysis (35, 36).

Na^+^/K^+^-ATPase is a transmembrane enzyme that consists of three subunits, α, β, and γ, where α is an integral plasma membrane protein that spans the membrane ten times and is the catalytic component, β is a glycoprotein that spans the membrane once, has an extracellular highly glycosylated domain and has a modulatory function, similarly to the small γ subunit, whose function has not been clarified so far (37). The phosphorylation of the catalytic subunit by kinases induced by different agents, including thyroid hormones and their analogs, represents a mechanism for its short-term modulation.

This type of regulation of the Na^+^ pump is mainly achieved by:1) Cyclic AMP that activates Protein Kinase A (PKA); 2) Diacylglycerol, endogenous activator of Protein Kinase C (PKC); 3) Phosphatidyl inositol 3-kinase (PI3K); 4) Intracellular Ca^2+^ increase and activation of Calmodulin Kinase (37, 38). It should be recalled at this point that the Na^+^/K^+^-ATPase is not only an important and ubiquitous pump that maintains the unequal distribution of ions across the plasma membrane, but it is also a “signal transducer” able to modulate important physiological responses such as growth, apoptosis, cell adhesion and migration (12). In this regard, the inhibition of the Na^+^ pump by ouabain and other cardioactive steroids is a way to modulate not only ion gradient across the plasma membrane, but also a possible pharmacological tool in case of cancer, viral infection and other pathologies (12, 38, 39).

## Thyroid hormones, 3,5-T_2_, Na^+^-dependent transport systems, cancer and immune function

In L-6 myoblasts thyroid hormones stimulate the Na^+^/H^+^ exchanger, a highly conserved integral plasma membrane protein that exchanges Na^+^ and H^+^ ions according to the concentration gradient. It does not require ATP hydrolysis, it is not electrogenic, exchanging two ions, Na^+^ and H^+^, in one to one ratio in opposite directions, but for its optimal functioning it requires the maintenance of the Na^+^ gradient by the Na^+^/K^+^-ATPase activity (8). Similar responses were found in chick embryo hepatocytes after treatment with T_3_ or 3,5-T_2_ (9, 13, 14).

3,5-T_2_, considered for years metabolically inactive, has been recognized in the last 20 years to activate a number of effects that are not only thyroid hormone-mimetic, but are instead independent of those of thyroid hormones. T_3_ and 3,5-T_2_ in chick embryo hepatocytes inhibited the Na^+^/K^+^-ATPase, stimulated the Na^+^/H^+^ exchanger, a signaling for DNA synthesis as well as amino acid uptake and intracellular calcium (Ca^2+^) release. The modulation of Na^+^/K^+^-ATPase and Na^+^/H^+^ exchanger determines an increase of intracellular Na^+^ content which, in turn, results in a modest depolarization of the cells, allowing the Na^+^/Ca^2+^ exchanger to operate in a reverse mode, further increasing intracellular Ca^2+^ levels, also for the inhibition of the Na-pump (40). This signal results in a mitogenic stimulation leading to cell proliferation and differentiation (13, 14). The inhibition of the activity of the Na^+^/K^+^-ATPase, studied by a pharmacological approach, was due to the activation of PKA, PKC, and PI3K (13, 14).

**Figure 1:**
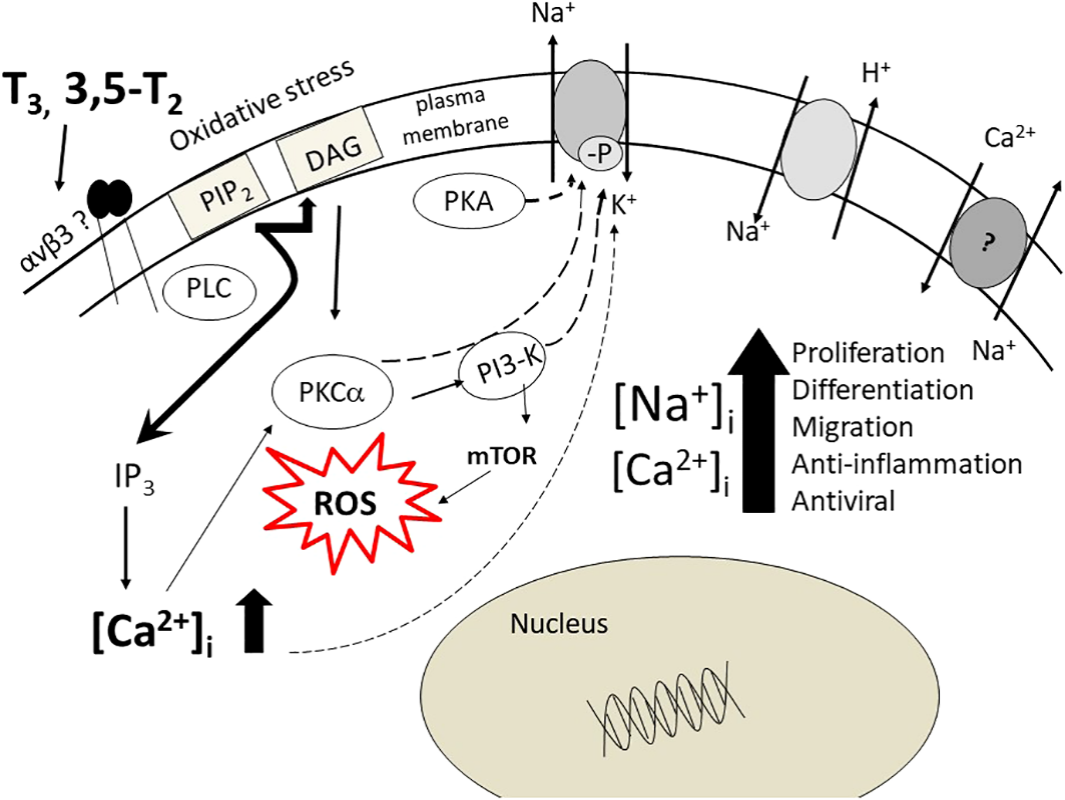
The pathways, cytosolic and nuclear, activated by thyroid hormone, T_3_, 3,5-T_2_ in chick embryo hepatocytes. Here a role for integrin αvβ3 is suggested. The increase of Na^+^ and Ca^2+^ ions is achieved by modulation of the Na^+^/K^+^-ATPase activity, the Na/H-exchanger, Na/Ca-exchanger (reverse mode). The final result is very similar to the activation of the α7nAChR as to increase of [Na^+^]_i_ and [Ca^2+^]_i_ ions and to cytosolic and nuclear pathways, resulting in the chick embryo in a coordinated response both mitogenic and immuno-defensive, aimed to the embryo survival. The figure is modified from Ref. 13.

As mentioned above, the chick embryo develops in an environment, the egg, separated from the maternal environment. The embryo starts to produce thyroid hormones at the time of pipping, close to term, and therefore thyroid hormones behave as a growth factor. They also support an immunological defense in dendritic cells in mammals (41, 42). Such an ionic environment, due to stimulation of the Na^+^/H^+^ exchanger and inhibition of Na^+^/K^+^-ATPase activity is typical of an anti-inflammatory response that may protect the chick at the time of hatching (**Figure 1**). A similar model was proposed for microglial cells under different physiopathological conditions: migration, adhesion, proliferation (43).

In human macrophages and in murine RAW 264.7 cells the treatment with ligands of the α7nAChR, the nicotinic receptor that mediates anti-inflammatory signaling, decreased TNF production following endotoxin treatment. Inhibitors or knockdown of the adenylyl cyclase 6 prevented the inhibition of TNF due to endotoxin, suggesting that the pathway of cAMP/PKA is involved in the immune response (44, 45).

T_3_ and T_4_, potentiate the antiviral and immunomodulatory effect of IFN-γ in HeLa cells, devoid of nuclear thyroid hormone receptor. The downstream pathway involves JAK1/JAK2 and tyrosine phosphorylation of STAT-1α and STAT-3, resulting in potentiation of EGF effect and, in the absence of EGF nuclear translocation, potentiation of tyrosine-phosphorylated MAPK (46–48).

We reported the capability of thyroid hormones to crosstalk with the immune system (41, 49) and to behave as anti-inflammatory agents in THP-1 human leukemic monocytes (50) and of 3,5-T_2_ (31).

The Na^+^/K^+^-ATPase inhibition has antiviral effects (51). Beside maintaining the electrolyte homeostasis of the cell, the Na^+^/K^+^-ATPase is also considered ‘a key scaffolding protein’ able to interact with other proteins elements of the signal transduction pathways, such as Protein Kinase A, Protein Kinase C, Phosphoinositide 3-Kinase (51–53). In particular, the inhibition of the Na^+^/K^+^-ATPase gives rise to an increase of intracellular Na^+^ and a decrease in K^+^. The second one is a signal for impairment of protein synthesis (53). Inhibition of Na^+^/K^+^-ATPase with ouabain or digoxin inhibits Zika virus infection in mice, while administration of extracellular K^+^ impaired the inhibitory effect (54, 55).

Interestingly the 3,5-T_2_ was ineffective in the modulation of Na^+^-dependent amino acid transport, whereas both T_3_ and T_4_ had a stimulating effect on the same transport system in chick embryo hepatocytes (25). A recent paper (56) pointed to molecular mechanism of Na^+^/K^+^-ATPase dysregulation as a main cause of alveolar epithelial barrier failure in severe Covid-19 infection. Indeed, Na^+^/K^+^-ATPase is the only transport system that eliminates Na^+^ from alveolar epithelial cells (57). In addition, Na^+^/K^+^-ATPase behaves as a cell adhesion molecule in epithelial cells and its abundance regulates the adsorption of the alveolar fluid, which drives the progression of acute lung injury (55, 57).

Na^+^/K^+^-ATPase is also a signaling molecule involved in the regulation of the intracellular Ca^2+^ concentration (58, 59), sensitive to oxidative stress (60, 61). Na^+^/K^+^-ATPase modulates actin cytoskeleton, cell volume and motility and interacts with growth factor/hormone receptors (62–64). Na^+^/K^+^-ATPase inhibition, by either ouabain or other cardiac glycosides (CG) as well as by 3, 5-T_2_ and T_3_, behaving as an ouabain-mimetic, contribute to impair viral activity. Convincing evidences indicate that viruses contribute to carcinogenesis (65). These observations highlight the pivotal role of Na^+^/K^+^-ATPase, beside the maintenance of the ion gradient, inhibited by 3,5- T_2_ and T_3_ as a potential anti-inflammatory, anti-cancer and anti-viral tool (13; **Figure 1**).

In fact, there are several aspects where the modulation of the Na^+^/K^+^-ATPase could be determinant and relevant to cancer growth. The interaction of T_3_, T_4_ and perhaps 3,5-T_2_, with integrin αvβ3 initiate a downstream signaling leading to modulation of PI-3K, MAPK and Ca^2+^ increase, as stated before. Resveratrol, a stilbene-derivative, is also a ligand of integrin αvβ3, not inhibited by Tetrac, gives rise through ERK1/2 activation, to accumulation of cyclooxygenase-2 (COX-2) and of tumor suppressor gene p53. Cyclooxygenase 2 (COX-2) is the rate limiting enzyme of the synthesis of the prostaglandins, induced by inflammatory mediators. The activation of ERK1/2 by thyroid hormone within 30 minutes elicits proliferation of glioma cells while PI3K activation increases the expression of hypoxia-inducible factor-1α (*HIF-1α*), as it comes from the activation of two different sites, S1 for *HIF-1α* transcription and S2 for cell proliferation on the integrin molecule (20, 66). *HIF-1α* stimulates the expression of vascular endothelial growth factor (VEGF) and angiogenesis, a determinant of cancer growth. MAPK is also related to the activation of STAT1α and is activated in turn by IFN-γ, an effect that, as stated above, is potentiated by thyroid hormone (48). STAT1 and STAT3 are downregulated by ouabain exerting anticancer activity (67, 68). The inhibitors of COX-2 in colon cancer increase the nuclear accumulation of p53. The induction of COX-2 is dependent upon p53-mediated activation of the MAPK pathway. These pathways are inhibited by ouabain (12, 69, 70). In line with this, the Na^+^/K^+^-ATPase is considered a target for the treatment of cancer and tissue fibrosis. In human lung fibroblasts (HLF) epithelial cells, and cancer associated fibroblasts (CAF) cardiotonic steroids, namely ouabain, blocked myofibroblast differentiation elicited by TGF-β. The effect was due to the inhibition of the Na^+^/K^+^-ATPase that gave an increase of Na^+^/K^+^ intracellular ratio, up-regulation of COX-2 and downregulation of TGF-β. The increased expression of COX-2 was abolished by inhibition of Na^+^/Ca^2+^ exchanger, indicating a role of Ca^2+^ signaling (71). The interaction of 3,5-T**_2_** with integrin αvβ3 has not been shown so far, but analogs of 3,5-T**_2_** such as sobetirome (GC-1) and Diiodothyropropionic acid (DITPA) have been shown to bind the integrin with activation of angiogenesis and MAPK, whereas the 3,5-T**_2_** activates PI3K improving insulin signaling in a model of NAFLD (72, 73, 74).

Of note, a primary energy-dependent Na^+^ efflux system is operative also in plants, although plant cells do not express a Na^+^/K^+^-ATPase like animal cells, but they do have an ouabain-sensitive Na^+^ pump (75). The ouabain sensitivity is maintained in the course of evolution and other growth factors or hormones such as Gibberellic acids (Gibberellins, GA) are used as plant growth regulators to stimulate both cell division and elongation that affect leaves and stems elongation as well as fruit ripening and flowering (76). Gibberellin in human cells increases the level of reactive oxygen species and protein apoptosis markers and GA inhibits the activity of the Na^+^/K^+^-ATPase and Ca^2+^-ATPase in human sperm (77).

## Conclusions

‘…. faciamus experimentum in corpore vili….’

The above-mentioned sentence was pronounced by an anonymous physician of the XVI century during a consulting among colleagues at the bed of the French humanist Marc Antoine Muret, (at that time in disguise and under poor clothes in Asti, Italy) that immediately was scared and felt suddenly healed….(Vocabulary -Treccani Institute).

So, overall, the physiological axis between thyroid hormones – 3,5-T_2_ and Na^+^/K^+^-ATPase offers promising perspectives from multiple point of views. In addition, research in the pharmacological inhibition of integrin αvβ3 might provide effective tools for cancer therapy as well as for the potentiation of immune system.

## Author contributions

Writing and original draft, SI and JZP: Writing – review and editing. All the authors read, made improvements, and approved the final draft.

## Conflict of interest

The authors declare that the research was conducted in the absence of any commercial or financial relationships that could be construed as a potential conflict of interest

## Publisher’s note

All claims expressed in this article are solely those of the authors and do not necessarily represent those of their affiliated organizations, or those of the publisher, the editors and the reviewers. Any product that may be evaluated in this article, or claim that may be made by its manufacturer, is not guaranteed or endorsed by the publisher.
